# Supplementary material for: Isolation and molecular characterization of Toxoplasma gondii from placental tissues of pregnant women who received toxoplasmosis treatment during an outbreak in southern Brazil
Source: PLoS One. 2020 Jan 30;15(1):e0228442. doi: 10.1371/journal.pone.0228442 (PMC6992202; doi:10.1371/journal.pone.0228442)
Supplement: S1 File — (DOCX) [file pone.0228442.s001.docx]

**Supporting Information file**

**Patient Bioassay 1.**

|  | Sinais clínicos | Dias de vida |
| --- | --- | --- |
| Mice 1 | apathy, bristly hair, photophobia, ascites | 12 |
| Mice 2 | apathy, bristly hair, photophobia, ascites | 12 |
| Mice 3 | apathy, bristly hair, photophobia, ascites | 11 |
| Mice 4 | apathy, bristly hair, photophobia, ascites | 13 |

**Patient Bioassay 2.**

|  | Sinais Clínicos | Dias de vida |
| --- | --- | --- |
| Mice 1 | apathy, bristly hair, photophobia, ascites | 13 |
| Mice 2 | apathy, bristly hair, photophobia, ascites | 14 |
| Mice 3 | apathy, bristly hair, photophobia, ascites | 15 |
| Mice 4 | apathy, bristly hair, photophobia, ascites | 12 |

**Pictures of bioassay mice showing some signs:**

**
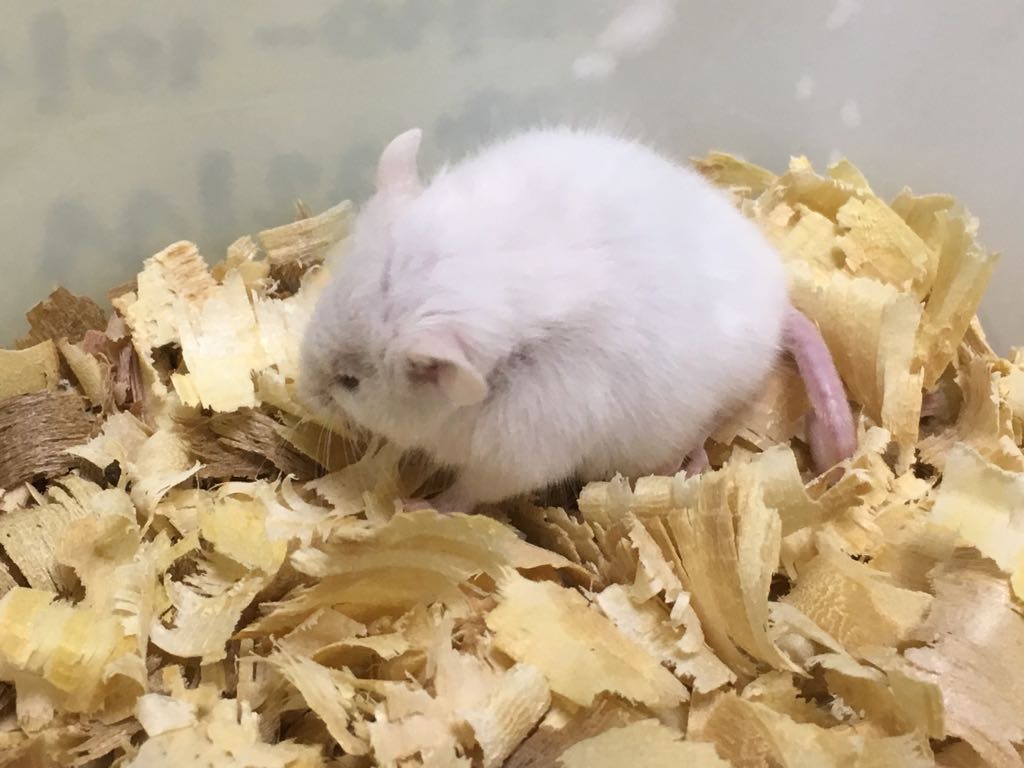
**

**
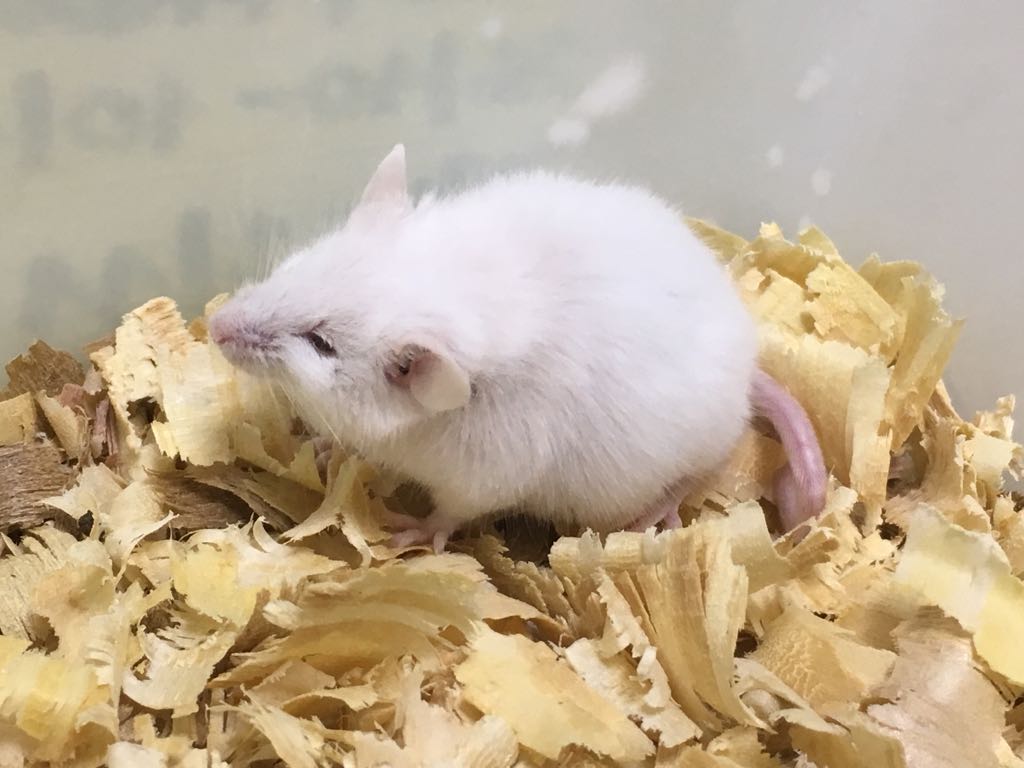
**
