# Supplementary material for: Isolation and molecular characterization of Toxoplasma gondii from placental tissues of pregnant women who received toxoplasmosis treatment during an outbreak in southern Brazil
Source: PLoS One. 2020 Jan 30;15(1):e0228442. doi: 10.1371/journal.pone.0228442 (PMC6992202; doi:10.1371/journal.pone.0228442)
Supplement: Attachment — Submitted filename: Reply to reviewers.docx [file pone.0228442.s002.docx]

**Reply to reviewers**

**Reviewer #1: This manuscript reported isolation and genetic characterization of Toxoplasma gondii strains isolated from congenital toxoplasmosis from an outbreak occurred in Santa Maria, Brazil in 2018, from which over 800 people were exposed to the parasite. Placenta tissues from two congenital toxoplasmosis were bioassayed in mice and the parasites were isolated from both cases. Genotyping by multilocus PCR-RFLP markers revealed a new genotype. The genotype seems to be highly virulent to mice.**

**The results of this study is of interest to molecular epidemiology of T. gondii, particularly for this largest known outbreak of toxoplasmosis in history.**

**The manuscript is overall well written, however, editing is needed to correct grammar errors.**

**There is a recent paper reported isolation of T. gondii in chicken from the same state (near Porto Alegre, Rio Grande Do Sul) (Andréia Vielmo et al., Outbreak of toxoplasmosis in a flock of domestic chickens (Gallus gallus domesticus) and guinea fowl (Numida meleagris). Parasitology Research, 2019, 118, 991–997). The genotype is almost identical to the one identified in this manuscript. The authors should look into the paper and discuss the results in that context.**

Reply:

Thank you for reminding us of this study. It will be added in our discussion.

**Specific Comments:**

**Line 33. It should be mice not rats.**

Reply:

Reviewed

**Lines 91-93. How much placenta tissue was used for pepsin treatment, 1 gram, 2 grams, or more? What was the volume the digested tissues were finally resuspended and then 1 ml was inoculated to each mouse?**

Reply:

50g of placental tissue were used for peptic digestion.

The digested material was resuspended in 5mL Saline.

**Use a table to summarize the results from mouse bioassay, including how many mice were inoculated, how many were positive with T. gondii and the days mice are euthanized.**

Reply:

Table 1. Mouse bioassay.

| Patients | Number of mice inoculated | Number of positive mice in the bioassay | Mice life days |
| --- | --- | --- | --- |
| **P. 1** | 4 | 4 | 11 - 13 |
| **P. 2** | 4 | 4 | 12 - 15 |

**Table 1. Was genotyping performed using DNA samples extracted directly from placentas? If so, list these samples in the table.**

Reply:

The table referring to genotyping will now be table 2 as another table has been included in the manuscript. Genotyping was performed only from the tissues of positive mice in the bioassay.

**Lines 162-164. It can be argued that the congenital infection has already occurred before the drug treatment, not necessary of the failure of the drug treatment.**

Reply:

Will be added to the text. Line 165.

**Lines 165-171. Given that the amount of parasites inoculated to mice is not known in this study, the result of high virulence could partially due to high inoculation does.**

Reply:

Will be added to the text. Line 173.

**Lines 173-175. The statement “It is necessary to consider a combination of alleles and formation of an atypical genotype may have contributed to an increase in virulence (Dardé, 2008)” is way beyond the scope of this study. Remove this sentence.**

Reply:

The sentence will be removed.

**Reviewer #2: The authors report isolation and molecular characterization of a strain isolated from placentas of 2 pregnant women infected during a large outbreak observed in 2018 in Rio Grande do Sul. The 2 isolates were identical with PCR-RFLP markers, suggesting a common source. This genotype seems different from the multiple ones already described in Brazil.**

**Although this report may be interesting as few isolates from human cases of toxoplasmosis are characterized in Brazil, it would have been more interesting if clinical data (mothers and babies) were made available to discuss the possible role of this genotype in the severity (or not) of clinical toxoplasmosis.**

**Apparently, the description of the large outbreak of Santa Maria is not yet published, and the paper presented here is just the contribution of the authors to the exploration of this outbreak. However, a few other information regarding isolates would be interesting. Are the 2 isolates described here the only ones available from this outbreak?**

Reply:

This study is really just a contribution to elucidating the causes of the great outbreak that occurred in Santa Maria. We agree that clinical data from patients would make the research more interesting, but not the central objective of the research. Thus we limit ourselves to isolation in mice and genotyping.

The 2 isolates described in this study come from the samples to which we had access. Thus we selected samples sent for diagnosis in our laboratory of patients who had received treatment for toxoplasmosis and who had children born alive.

- **L. 55-56: “In Brazil …. isolation and genetic characterization of T. gondii from human tissues, especially from pregnant women, are scarce.”: yes, but they do exist. Could the authors add Brazilian references, at least for congenital cases? (Carneiro et al., 2013 J. Clin. Microbiol. 51, 901–907; Ferreira et al., 2011 Exp. Parasitol. 129, 190–195; Silva et al., 2014 PloS One 9, e90237). The authors could discuss about the comparison with other strains isolated from congenital cases in Brazil.**

Reply:

Thank you for the indication of the studies. They will be added to the manuscript.

**- L.84: “the diagnosis of the two pregnant women occurred in the final trimester of gestation.”: does that mean that infection occurred in the last trimester or just that the diagnostic was made at this stage?**

Reply:

We cannot say when the infection occurred. Only that the diagnosis was made at this stage.

**- L. 145: “Detection of protozoal DNA in mouse tissues also indicates the feasibility of T. gondii infection in placental tissues.”: do you mean the presence of Toxoplasma infection in placenta, not the feasibility?**

Reply:

Exactly. We refer to the presence of Toxoplasma in the placenta. This sentence will be corrected in the manuscript.

- **Genotype: the authors described the genotype obtained as a new genotype for Brazil, according to ToxoDB. It would be interesting to show a dendrogram indicating the place of this genotype among other genotypes described in Brazil. Or at least, add to table 1, the PCR-RFLP profiles of the main Brazilian genotypes (Br I to IV) and other genotypes found in Rio Grande do Sul state in animals.**

Reply:

A table with this data will be added to the discussion of the manuscript.

Table 3: Genotypic characterization of T. gondii isolates obtained from two patients during the Santa Maria toxoplasmosis outbreak compared to three other isolates [9,28].

| Isolado | Markers | | | | | | | | | | | |
| --- | --- | --- | --- | --- | --- | --- | --- | --- | --- | --- | --- | --- |
|  | Sag1 | 5’Sag2 | 3’Sag2 | Sag3 | Gra6 | BtuB | C22-8 | C29-2 | L358 | PK1 | Alt.SAG2 | Apico |
| **^a^ P. 1** | I | I | I | I | III | III | II | III | III | I | I | I |
| **^a^ P. 2** | I | I | I | I | III | III | II | III | III | I | I | I |
| **^b^ Vielmo et al.** | I | I | I | I | III | III | II | III | III | I | I | III |
| **^c^ BrI** | I | I | I | III | II | I | u-1 | I | I | I | I | I |
| **^c^ Br IV** | I | III | III | III | III | III | II | I | III | III | III | III |

This is the Table 3 legend.

**^a^** Outbreak patients isolates

^b^ Isolated recently described in Rio Grande do Sul

**^c^** Common isolates in Brazil

**- L.160: “This result is uncommon, since only a few researchers have been able to carry out the T. gondii isolation from pregnant women tissues,”: this is not true and reports of isolation of T. gondii form placenta are very common (see for example, Ajzenberg et al. JID 2002; Robert-Gangneux et al. 2010)**

Reply:

This sentence will be corrected in the manuscript.

**Reviewer #3: There are articles with the molecular characterization of approximately 50 T. gondii strains , it seems to me very little to publish an article with the characterization of 2 strains.**

**There are more recent methodologies for molecular characterization such as microsatellites and NGS.**

**Isolation of T. gondii strains from biological samples such as amniotic fluid placentas and newborn blood by inoculation in mice is a routine and ancient procedure used by European reference laboratories.**

Reply:

We understand that if the number of isolates surveyed were larger, the study would be more interesting. However, we are limited to the number of samples that have arrived at our diagnostic laboratory. Unfortunately we could not include more isolates in our search.

Regarding the technique used, we chose RFLP because it is a reliable technique widely used by researchers in the field. Besides being the technique available in our laboratory.

We know our research has limitations, but we are dealing with samples from what appears to be the largest outbreak of toxoplasmosis ever described in the world. This makes us think that although we have had access to few isolates, the results of isolation and genotyping are interesting and cannot be ruled out. With this we intend to make a small contribution to the elucidation of the factors that contributed to the occurrence of this outbreak.
