# Supplementary material for: Isolation and molecular characterization of Toxoplasma gondii from placental tissues of pregnant women who received toxoplasmosis treatment during an outbreak in southern Brazil
Source: PLoS One. 2020 Jan 30;15(1):e0228442. doi: 10.1371/journal.pone.0228442 (PMC6992202; doi:10.1371/journal.pone.0228442)
Supplement: Attachment — Submitted filename: Reply to reviewers.docx [file pone.0228442.s003.docx]

**Reply to reviewers**

**Reviewer #1:**

**Line 186. Suggest revise to “ … capable of causing a chicken outbreak on a small rural property, suggesting these two closely related genotypes are virulent to humans and animals.”**

Reply:

Thank you for the consideration. The sentence has been changed in the manuscript.

**Reviewer #2:**

**Abstract: “In addition, the samples were positive in conventional PCR and, when submitted to PCR-RFLP genotyping, presented an atypical genotype,…”: this sentence is ambiguous, since PCR-RFLP was not performed directly on placenta DNA extract but on the strain isolated in mice.**

Reply:

Thank you for the consideration. The sentence has been amended in the abstract to improve understanding.

**L. 104: “In all cases, 20mg of tissues were used for DNA extraction”. For placenta, was it 20mg of tissues or 20 mg of the pellet obtained after peptic digestion?**

Reply:

For extraction of placental DNA, 20mg were used directly from the placental tissue.

**L. 191: “both genotypes differ from the Brazilian clonal lineages, BrI, BrII, BrIII and BrIV [9], as shown in table 2”: only Br1 and BrIV are presented in table 2. BrII and III should be added to table 2.**

Reply:

BrII and III strains were added to the table.

**Ref 19: not accessible, the link does not work.**

Reply: The link has been changed.

**The text contains many errors in English that need to be corrected.**

Reply:

The text has been subjected to a new English revision. We hope we have managed to improve the language.
