# Supplementary material for: Isolation and molecular characterization of Toxoplasma gondii from placental tissues of pregnant women who received toxoplasmosis treatment during an outbreak in southern Brazil
Source: PLoS One. 2020 Jan 30;15(1):e0228442. doi: 10.1371/journal.pone.0228442 (PMC6992202; doi:10.1371/journal.pone.0228442)
Supplement: Attachment — Submitted filename: Response to Reviewers.docx [file pone.0228442.s004.docx]

**Response to Reviewers**

**Page 4, lines 72-73 please replace "who who delivery babies their at the University Hospital" with "who delivered their babies at the University Hospital"**

**Page 6, line 135 please replace "T. gondii wes isolate" with T. gondii was isolated"**

**Page 8, line 165 please replace "Santa Maria, was isolated T. gondii from placental tissues" with "Santa Maria, T. gondii was isolated from placental tissues"**

**Page 9, line 175 please replace "art characteristic" with "are characteristic"**

**Page 9, line 185 "the corrent" should be "the current"**

Response:

Thank you very much for the considerations. All of them were made in the manuscript.
